# Acceptability and experience of a personalised proteomic risk intervention for type 2 diabetes in primary care: qualitative interview study with patients and healthcare providers

**DOI:** 10.1017/S1463423621000591

**Published:** 2022-04-01

**Authors:** Stephanie Honey, Richard D. Neal, Michael Messenger, Samuel G. Smith

**Affiliations:** 1 Leeds Institute of Health Sciences, University of Leeds, Leeds, UK; 2 Leeds Centre for Personalised Medicine & Health, University of Leeds, Leeds, UK

**Keywords:** behaviour change, proteomic risk assessment, pre-diabetes, type 2 diabetes

## Abstract

**Aim:**

We explored the acceptability of a personalised proteomic risk intervention for patients at increased risk of type 2 diabetes and their healthcare providers, as well as their experience of participating in the delivery of proteomic-based risk feedback in UK primary care.

**Background:**

Advances in proteomics now allow the provision of personalised proteomic risk reports, with the intention of achieving positive behaviour change. This technology has the potential to encourage behaviour change in people at risk of developing type 2 diabetes.

**Methods:**

A semi-structured interview study was carried out with patients at risk of type 2 diabetes and their healthcare providers in primary care in the North of England. Participants (*n* = 17) and healthcare provider (*n* = 4) were interviewed either face to face or via telephone. Data were analysed using thematic analysis. This qualitative study was nested within a single-arm pilot trial and undertaken in primary care.

**Findings:**

The personalised proteomic risk intervention was generally acceptable and the experience was positive. The personalised nature of the report was welcomed, especially the way it provided a holistic approach to risks of organ damage and lifestyle factors. Insights were provided as to how this may change behaviour. Some participants reported difficulties in understanding the format of the presentation of risk and expressed surprise at receiving risk estimates for conditions other than type 2 diabetes. Personalised proteomic risk interventions have the potential to provide holistic and comprehensive assessments of risk factors and lifestyle factors which may lead to positive behaviour change.

## Introduction

An estimated 12.3 million people in the UK are at high risk of developing type 2 diabetes (Diabetes UK, 2020). People with type 2 diabetes experience complications, including cardiovascular and kidney disease. A recent study reported that finding and managing patients at high risk of type 2 diabetes could prevent cardiovascular disease (Cai *et al.*, 2020). Treating and caring for type 2 diabetic patients costs an estimated £8 billion per year in the UK (Diabetes UK, 2020). However, over half of type 2 diabetes cases can be prevented by lifestyle changes (Diabetes UK, 2020). People at increased risk of type 2 diabetes in England are invited to attend the National Diabetes Prevention Program (NDPP) or other, locally provided, education programmes. The NDPP educates and supports lifestyle behaviour change, including weight loss, physical activity and improved nutrition to prevent the conversion to diabetes.

A recent report evaluating early outcomes of the English NDPP reported that participants achieved reductions in their HbA1C results and lost weight, potentially decreasing the number of people who will develop type 2 diabetes (Valabhji *et al.*, 2020). This reflects the findings of a previous meta-analysis concluding that diabetes prevention programmes, when compared with usual care, could significantly reduce the development of type 2 diabetes in high risk populations (Public Health England, n.d.). Internationally, research on diabetes prevention programmes have come to similar conclusions (Knowler *et al.*, 2002; Ramachandran *et al.*, 2006; Tuomilehto *et al.*, 2009). The uptake of the English NDPP programme, however, is poor. Almost half of patients referred decline to participate and only one-fifth, of those referred, complete more than 60% of sessions (Valabhji *et al.*, 2020).

Providing personalised information to individuals at higher risk of type 2 diabetes may increase willingness to change behaviour. Attempts to achieve behaviour change through the provision of personalised genetic information for a range of conditions have largely been unsuccessful (Hollands *et al.*, 2016). Proteomic-based risk assessments may offer a new opportunity for supporting behaviour change in patients, but they have not been tested in clinical practice. Proteins capture both genetic and environmental influences and reflect the real-time health status of the individual (Suhre *et al.*, 2017). Presenting such information when providing risk feedback to patients may therefore encourage greater engagement. SomaLogic Inc. have developed a method of attempting this; their ‘SOMAscan Insight Report’ presents individual proteomic results in a user-friendly patient-centred format (Somalogic, n.d). We refer to this as a ‘personalised proteomic risk report’ and, when combined with the healthcare provider feedback, as the ‘personalised proteomic risk intervention’. At present, there is no evidence that such an approach works, so we tested the intervention among patients at high risk of type 2 diabetes. We chose this patient group because of their increasing levels of clinical need and their low levels of engagement with existing services (Barron *et al.*, 2018).

In this study, we explored the acceptability of the personalised proteomic risk intervention for patients at increased risk of type 2 diabetes and their healthcare providers, as well as their experience of participating in the delivery of the personalised proteomic risk intervention in UK primary care.

## Method

### Setting

A qualitative semi-structured interview study, nested in a single-arm pilot trial, was undertaken in primary care in Leeds between May 2019 and March 2020.

### The intervention

**Figure 1.**
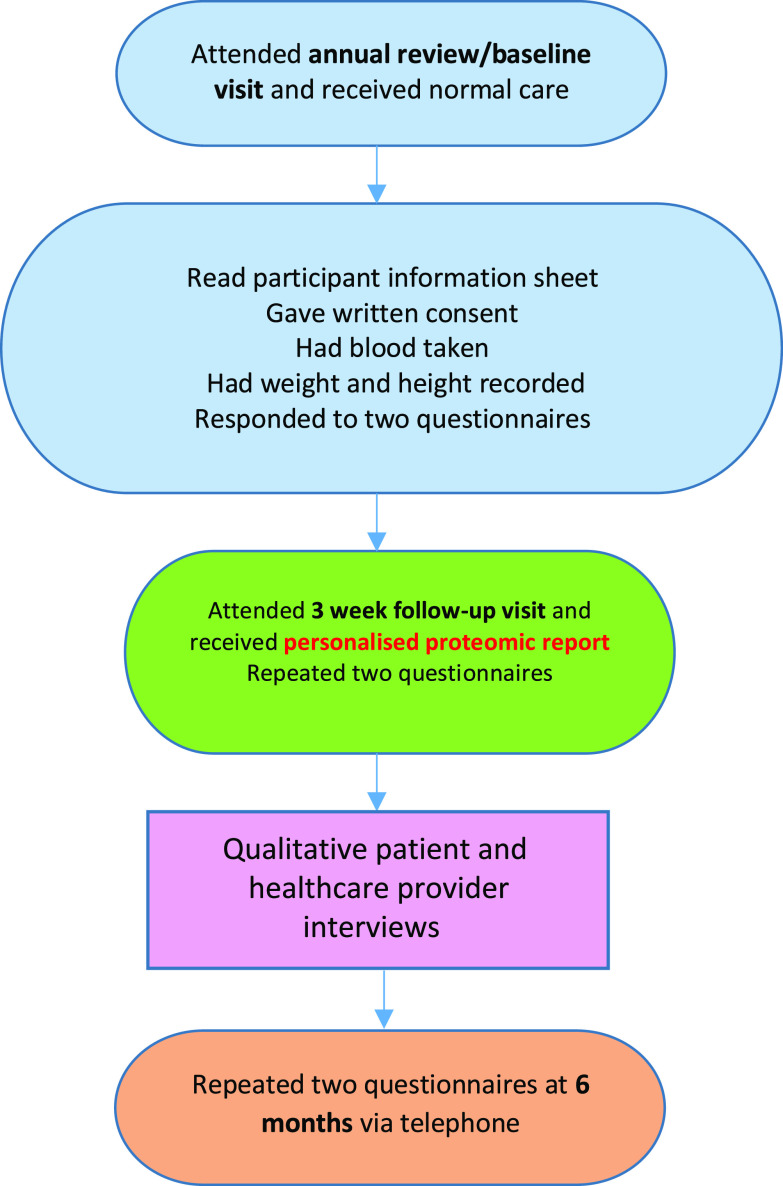
Trial processes for ACTIVATE participants.

The intervention consisted of the personalised proteomic risk report being presented to the patient 3 weeks after their baseline appointment (see Figure 1). The report, a nine-page A4 printout, was presented by a healthcare provider in an extended face-to-face consultation. The report included personalised, detailed, information about the patient’s risk of diabetes and heart, kidney and liver disease. Information about their lifestyle, such as smoking and alcohol consumption, were also presented. The patient was given the opportunity to discuss their report with the healthcare provider. They were given appropriate advice and support to encourage and enable any necessary behaviour change. There was also space on the report for the patient to take notes and to develop an action plan with the healthcare provider. The contents of the report are further detailed in Box 1, and an example report is given in Appendix 1. This intervention included several of the ‘intervention functions’ recommended in Michie *et al.*’s Behaviour Change Wheel (2011). These included incentivisation, education, persuasion and enablement for change. Hence, the intention was that the patient would reflect on their report, link their identified risks with their lifestyle and be motivated to change their behaviour with the support of their healthcare provider (ibid).

Box 1. Contents of the report

The report took the form of an A4 paper booklet that provided a personalised report of:

- Diabetes risk (with indicators pointing to ‘typical’ or ‘increased’).
- Heart disease risk (low, medium or high).
- Kidney function (no damage or some damage).
- Liver fat (no fat build-up or liver fat).
- Percentage body fat (less than average, average and more than average).
- Lean body mass (less than average, average and more than average).
- Tobacco smoke exposure (no exposure or some exposure)
- Alcohol use (below or above 14 units).
- A summary page, on which the patient could make notes.
- A diabetes prevention action plan grid, on which the patient could write their goals.

### Recruitment

Participants were recruited for this nested semi-structured interview study at the baseline study visit. Seventeen patient participants were recruited from seven GP practices. Purposive sampling of practices was attempted to give a range of geographic locations, socio-economic information and uptake of the NDPP. In practice, very few practices were interested in participating, so a convenience sample was used. Purposive sampling of patients was used to elicit the views of a range of ages (42 years old to 82 years old) and genders (4 women and 12 men). Unfortunately, only White patients agreed to be interviewed. Eligible patient participants were at high risk of diabetes as defined by having had an HbA1c between 42 and 47 mmol/mol recorded within the last 12 months, aged 40 years or over, attending their GP practice for annual pre-diabetic reviews and had previously declined to participate in the NDPP. Patient participant recruitment ceased at the point data saturation was reached. Healthcare providers involved in conducting baseline and follow up visits with at least three participants were eligible to participate. Sixteen healthcare providers were invited to participate and four agreed, giving a response rate of 29%. They comprised one practice nurse, two research nurses and one healthcare support worker (see Table 2.) The two research nurses had assisted with the research tasks when practice staff were experiencing high workloads. They carried out most of the baseline and 3-week follow-up visits for two practices. The ACTIVATE trial processes are shown in Figure 1.

### Interview process

Interviews were conducted face to face or via telephone. Participants were told the researcher (SH) was employed by the University of Leeds and was a nurse by background. SH conducted all the interviews. The healthcare provider interviews took place in person at GP surgeries, the University of Leeds or via telephone. Topic guides were developed following consultation of the scientific literature and discussions between the research team (RN, MM and SS), the ACTIVATE steering group and a patient and public involvement group. Written or electronic consent was obtained from all participants. The personalised proteomic risk report (as detailed above) was used as a prompt in both the participant and healthcare provider interviews.

### Analysis

Analysis was carried out by SH and SS who have experience in qualitative research. Interviews were digitally recorded, transcribed verbatim and anonymised. Transcripts were read to gain insight into patient and healthcare provider experiences and coded by SH using NVivo (V12). Codes were developed iteratively, and the coding structure and code reports were verified and discussed with SS and RN (Pope *et al.*, 2000). The transcripts were interrogated to find repeated patterns of meaning (Braun and Clarke, 2006). This allowed the researchers to examine the experiences, meaning and reality of the participants involved in the trial (Patton, 1990). Important themes were compared with others within and across transcripts (Glaser and Strauss, 2009). The themes were discussed between three researchers (RN, SS and SH) and named before secondary coding was undertaken (Richards, 2005). Final interpretation of the data involved studying the themes, the links between the themes and relating these findings back to the research questions and relevant literature (RN, SS and SH). COREQ guidance on the reporting of qualitative data was adhered to (Tong *et al.*, 2007). Patient confidentiality was maintained at all times.

## Results

### Participant characteristics

Seventeen patients were interviewed (Table 1). Fourteen interviews were conducted face to face in the patients’ homes and three by telephone. The average duration was 48 min. Four healthcare providers were interviewed. Two interviews were conducted at their practice and two at the University of Leeds (Table 2). The average duration was 56 min.

**Table 1.** Participant characteristics

ID	Sex	Age in years	Ethnicity	Interview type
6F	F	65	White	Face to face
08	M	70	White	Face to face
9BF	F	51	White	Face to face
BF	M	71	White	Face to face
C6	M	70	White	Face to face
FB	M	70	White	Face to face
85	M	72	White	Face to face
FC	M	68	White	Face to face
AD	M	43	White	Face to face
D8	M	42	White	Face to face
9BE	M	82	White	Face to face
D1	M	72	White	Face to face
E2	M	66	White	Face to face
7A	F	70	White	Face to face
CB	F	65	White	Telephone
1D	M	70	White	Telephone
A2	M	78	White	Telephone

**Table 2.** Healthcare provider characteristics

ID	Sex	Age in years	Ethnicity	Role	Years’ experience	Interview type
S1	F	47	White	Practice Nurse	29	Face to face
S2	F	22	White	Healthcare Assistant	1	Face to face
S3	F	57	White	Research Nurse	30	Face to face
S4	F	61	White	Research Nurse	43	Face to face

### Themes

#### Understanding of the personalised proteomic risk report

The patient’s individual risks of developing type 2 diabetes, and related conditions, were discussed using their personalised proteomic risk report. Most patients found the format of the report clear and easy to understand. They also appreciated having the report, with all the results in together, to take home. One patient, for example, reported being impressed by the format and analytic content of the report and compared it to more generalised advice:

This [the report] is a different thing, this is where somebody is giving you factual information, it’s been analysed by a computer…and it just works it all out and then it has presented you with the facts, that’s it. It is better. I must admit, it’s a good idea. (FB, 70 years. M)

Patients also appreciated the personalised nature of the report, and that the results for all aspects of their health were presented together:

These [results] are more personalised than usual and in-depth. It’s good to understand that other parts of the body are influenced by diabetes, and they can do checks on the kidneys, for instance, and your liver. That’s a very useful thing, a general everything sort of thing. (E2, 66 years, M)

In addition, some patients enjoyed the longer appointments:

I understood the report fine…It was also really good to have a double appointment or whatever it was, well, it was longer than usual anyway. Time to discuss things properly. (AD, 43 years, M)

Some patients, however, would have preferred additional, more detailed, information to help them interpret their results:

I found it [the report] a little…I don’t want to say simplistic, but it almost seems a little bit abstract: ‘Heightened/not heightened/medium’, I’m not sure what that means’. (D8, 42 M)

Most patients understood their individualised risk of developing type 2 diabetes and knew that the risk, as it was presented, was relative to the population known to be at risk of type 2 diabetes, and not the general population:

Yes, no worries about that [the information provided], that was fine…I’m three times as likely to develop it as other people in the prediabetic range. (CB, 65 years, F)

A minority of patients were not aware of this subtlety and thought the risk, as presented, was relative to the general population:

No, I didn’t know that [diabetic risk] was for people who had already been told they were prediabetic. Not until you [interviewer] just said, anyway (08, 70 years. M)

The healthcare providers were happy with the format of the report and thought it was generally easy to understand. Similar to patient ‘08’, one healthcare provider felt diabetes risk could have been presented more clearly:

You are actually talking about a ‘typical risk’ or an’ increased risk’ for somebody who is already prediabetic and it’s that patient population that you are being compared against not the general population, and unless that’s very clearly put across to the patient [by] healthcare providers doing this, the patient’s answer will be, ‘Well I know I’m at risk, that’s why I’m here in the first place’. (S1, F, practice nurse)

The healthcare provider also agreed that more information would have been useful in some instances:

I think for the heart disease risk, ‘low’, ‘medium’ and ‘high’, I think that [the presentation in the report] is good…I think the kidney function where it was either ‘no damage’ or ‘some damage’ is quite ambiguous really. What do you mean by some damage?…It’s easy if it’s no damage, but when it becomes some damage it’s like what level? I think you need more of a continuum on that. (S1, F, practice nurse)

#### *Risk perceptions* and *motivation*

It was evident that many patients understood the subtleties of their risks, as presented in their reports and this gave them motivation to change:

When I saw the diabetic one [result] I thought ooh great, like not at high risk, but then I thought ‘but I’m still prediabetic but not at increased risk’. It’s typical risk for being borderline…It’s made me think about it more, everything, diet and exercise. I don’t want to be one of these that’s on all these tablets. (6F, 66 years. F)

Some patients, however, downplayed the importance of their risks and were less keen to make lifestyle changes. Several participants regarded increased risk as a natural consequence of ageing and were sometimes sceptical or frustrated by lifestyle advice:

[I have been prediabetic] for two or three years – but I’m not a diabetic yet. Yes, it’s probably change of lifestyle with the onset of retirement and age really, natural progression. There’s so many aspects to it these days and the press love to get hold of it and what’s in favour one week is out the next. You’ve got to make your own decisions really. (08, 70 years. M)

There was some indication that worry might be increased through the disclosure of risks and abnormal results:

I seem consistently prediabetic, with a little nod into diabetic…I’m three times as likely to develop it [type 2 diabetes] as other people in the prediabetic range… [The heart disease risk] was a bit of a new thing. I’d kind of guessed, due to being overweight and unfit, so I figured I was heading that way… I was worried about that and the kidney damage. It’s hard actually trying to make the changes. (D8, 42, M)

One healthcare provider said that the manner in which the report was presented, as well as its format and contents, enhanced patients’ perceptions of risk and favourably influenced their motivation to change:

The fact it’s [the report] is presented to them is good. Because normally when you have a blood test…you only hear back if it’s abnormal. Normally they don’t get a sit-down appointment. So it’s that consolidation of it face to face. I will try and stress the importance of it, but say ‘This is something you can deal with, it can be managed, and that’s the good thing about this: it’s telling you what your risk is and now you can start doing something about it’. That motivates them. (S4, F, research nurse)

Another healthcare provider agreed that the report provided motivation:

People get inured to the bog-standard ‘you must diet’ [and so on]. They’ve got fed up of hearing it. So this is something new…and it’s caught their attention. They feel privileged and motivated to be part of the future (S3, F, Research nurse)

#### Results showing conditions patient were unaware of

While some patients were alarmed by unexpected, abnormal results, they felt it was useful to have found out so action could be taken:

I was surprised with [the liver fat result] because of late I’ve not been drinking half as much for the past year or so, I’ve cut my drinking down and everything. I imagined it would be quite a healthy liver…I do exercise more and watch what I eat nowadays and not take as much…not stopped drinking but cut my drinking down, take exercise. (C6, 70 years. M)

Some patients were not concerned about unexpected results, possibly due to reassurance provided by the healthcare provider:

Well, you don’t know what goes on inside your body, do you? If it’s showing some kidney damage but I’m still functioning normally, then I wouldn’t worry about it. Obviously it was worth checking out again and the doctor said it’s nothing to worry about. (A2. 66 years, M)

Other patients had unexpected results but reported that they were not reassured or given any advice:

That’s the one that interests me the most, the liver fat…I would be interested to find out how bad it is…because I’d like to try and do something…Obviously I’m still doing something that is causing it. They [healthcare providers] didn’t say anything about it though. They didn’t tell me how to get rid of it (CB, 65 years, F)

One healthcare provider reported that most patients she had seen preferred to know about such results but she tried to ensure this did not cause undue worry:

Nearly all of them [patients] reacted very favourably, even if they ended up with a surprise result, they said they’d rather know and be able to do something about it…And also I ask them if they were worried by it, because I don’t want them to go away worried, and most of them didn’t seem to be. Obviously they’d rather it wasn’t that. (S4, F research nurse)

#### Body fat, lean body mass, tobacco exposure and alcohol intake results

Patients were impressed with these ‘lifestyle’ aspects of their results. Patients who had good results were pleased that their behaviour change efforts were proving to be worthwhile:

I was really pleased [with results]…I loved my muscle ratio to fat…it has reassured me, that my running is working…When you are slogging up that hill, it really is hard, but yeah, it makes it worth it. I said to Doctor [name], ‘How on earth do they know this from a blood test?’… It is incredible! (9BF, 51 years. F)

It [the report] says I’ve got more than average fat – 38% fat. If I lose a couple more stone then I would be down to normal. And my lean body mass, I’ve also got more than average, so that’s good because that means I’ve got muscle. That must be the muscle I have been building on my exercise bike [laughs] so I will keep on doing that (FC, 68 years, M).

#### Behavioural responses to the personalised proteomic risk intervention

Many patients, deemed to be at ‘typical risk’ for diabetes, were reassured and motivated to continue their efforts to lose weight and take more exercise:

I am still in the prediabetic range. I would love to get that number down and I still have got some work to do. Hopefully, by the time I get my next set of blood tests next year, I am hoping that I can get my HbA1c at least below the 42…So, I am going to keep on with the exercise, keep on with the weight loss, keep watching my diet and hopefully, I can reduce it, so that I am no longer prediabetic. That is the idea. (9BF, 51years F)

Some patients at ‘increased risk’ also said the report had motivated them to change:

I have had the warning; it’s [his results] given me a warning to mend my ways or else. I need encouragement. Everybody does. Short sharp shock type thing to make you get off your backside and do something. It is having the motivation, isn’t it?…It’s made me concerned about what I eat and how much I drink, what exercise I take. I have been walking more and only drink at weekends now, if there is nothing special on. (C6, 70 years. M)

Since getting it [the report], we [patient and his wife] are trying to keep our sugar levels down…we have found different ways of eating, trying different ways of cooking…We are cutting out as much fat as we can. (AD, 43 years. M)

Other patients at ‘increased risk’ were less concerned and were not as strongly motivated to change their behaviour:

I was quite happy [with the report] really. It didn’t worry me. Basically, I just thought it [risk of diabetes] were a thing that comes to you with age…It wasn’t something to worry about. I think it depends on your make-up. My uncle Joe…had never smoked…he died of cancer. I am not stopping my latte coffee! I would finish up I wouldn’t be eating anything. When you get into your 80s, what else is there to enjoy? (9BE, 82 years, M)

A diabetes prevention action plan grid, on which the patient could write their goals, was included in the report booklet (see page 8 of the report, Appendix 1.) Only one patient interviewed reported being asked to fill these sections in. When this was mentioned by the researcher to one healthcare provider she was surprised:

Why are they [healthcare providers] not doing the action plans?…that’s astounded me! They should be doing that…We all know the research proves that people only absorb so much and retain so much, so the best practice is to give them something written to go home with. One lady I saw [who had made a plan with the nurse], she’s going to go out and do some salsa dancing and some other exercises and get her stress levels down. A plan will work. (S3, F, Research nurse)

The report did not appear to motivate uptake of the NDPP, with no participants reporting attendance following their personalised risk results.

## Discussion

### Summary of main findings

This is the first study to report the acceptability and experience of using a proteomic assessment for type 2 diabetes risk in primary care. The personalised proteomic risk report used in this study is unique in the way it provides the most comprehensive picture of the human proteome available to date (Somalogic n.d). Proteomics are influenced by individual lifestyle behaviours (Suhre *et al.*, 2017), and therefore this higher level of personalisation may encourage greater engagement with diabetes prevention. Ensuring this approach is acceptable and that the patient experience is positive is a necessary first step towards implementation in routine practice.

Participants mostly found the intervention an acceptable approach to providing diabetic risk information and reported a positive experience. As a consequence of the personalised proteomic risk intervention, patients reported a high level of insight and understanding about their increased risk for diabetes, as well as their risk of associated comorbid conditions. Other factors contributing to the positive experience include that the results were presented in a longer face-to face consultation than usual, and using a format that was different to participant’s previous experience. A minority of participants misunderstood the risk feedback, as they did not realise it was framed in relation to other people with pre-diabetes and not the general population. Healthcare providers also acknowledged that the report could have been clearer in places, and that caution may be needed when using labels such as ‘typical risk’ in the report. Participants regarded the test as novel and felt privileged to be taking part in research.

While the study was not designed to ascertain the impact of the personalised proteomic risk intervention on subsequent lifestyle behaviours, we did identify some signals that the test could be useful in this regard. Participants perceived their risk to be higher following the report, a construct known to be key in motivating behaviour change (Sheeran *et al.*, 2014; Ferrer and Klein, 2015). Furthermore, several participants reported specific behaviours that had improved following the report, including limiting alcohol intake, and improved diet and physical activity. The report did not appear to motivate uptake of the NDPP.

### Strengths and limitations

This study used robust qualitative methods, providing an in-depth exploration and understanding of patient experience and acceptability of a novel test. Limitations of the work include a lack of ethnic diversity in our patient sample, recruitment from a single centre and a likely lack of saturation in our healthcare provider sample. The cross-sectional design did not permit any assessment of the effect of the intervention on actual behaviour change.

### Comparison with existing literature

Our findings suggest some participants at high risk of developing diabetes could be motivated to change their behaviour by engaging with the intervention. This is in contrast to the findings of a recent systematic review that suggested genetic risk communication did not lead to lifestyle change (Hollands *et al.*, 2016). The review did, however, suggest that genetic risk communication may have some influence if effective behaviour change interventions are provided alongside the provision of results. It may be that patients in our study were frustrated by the usual monitoring and advice being given, and that healthcare providers had also become less inspiring in its delivery. A completely new way of presenting risk, along with lifestyle information, may be more likely to motivate patients. It is possible that the provision of personalised, proteomic risk assessments and interventions supplemented with ongoing support in primary care could help people at high risk of developing diabetes adopt and maintain positive health behaviours.

Our study found that many participants were not interested in attending an NDPP session. This denied them the potential benefits of such participation which could include weight loss, a reduction in their HbA1c levels, and possibly, a reduction in their likelihood of developing type 2 diabetes (Valabhji *et al.*, 2020; Public Health England, n.d.). This mirrors the findings of a new report which estimated only 36% of eligible patients attended an NDPP educational session (53% attended an initial assessment) (Valabhji *et al.*, 2020). A literature review and behavioural analysis of the uptake and retention of group weight management programmes found that people with knowledge were more likely to enrol on such programmes (Public Health England, n.d.). The personalised, proteomic risk report could provide individuals with this knowledge which may encourage participation in the NDPP.

Some patients in this study appeared to be more interested in online programmes and the NDPP are planning to offer such sessions in the future. There is evidence that online programmes can help patients with lifestyle change. A review of lifestyle interventions based on the diabetes prevention programme delivered digitally was promising (Joiner *et al.*, 2017). This study found, however, that the programmes that also included the support of counsellors were most effective. It may be that a comprehensive, face-to-face consultation, where patients receive their reports, followed by ongoing motivational tools accessed online, could successfully support patients’ lifestyle change efforts.

All but one patient in our study reported that they were not encouraged to fill in the goal setting grid provided at the end of the report. This could have been a missed opportunity as there is evidence that action planning can help motivate patients to achieve their heathier lifestyle goals (Rhodes and Dickau, 2012; Hagger and Luszczynska, 2014). Patients need to select feasible goals and have a concrete plan to put them into action (Bailey, 2017). One study demonstrated how action planning can be implemented in primary care (Handley *et al.*, 2006). Patients with coronary heart disease identified goals to improve their health such as becoming more physically active. Over half of the patients achieved their goals even though the goal setting sessions were only 7 min in duration on average. Being told simply to exercise more and lose weight, without a plan in place, may fail to motivate patients.

### Implications for policy, practice and research

This study has shown that a personalised proteomic risk intervention is acceptable to staff and patients and that it is feasible to implement this novel technology into primary care in the UK. The personalised proteomic risk report could be used for a wide variety of conditions or general wellness checks. Further research could include an in-depth investigation into patients’ responses to receiving lifestyle information (e.g. body fat percentage plus tobacco exposure) along with results pertaining to medical conditions and how this affects motivation to change behavior.
